# Does pronator quadratus repair affect functional outcome following volar plate fixation of distal radius fractures? A systematic review and meta-analysis

**DOI:** 10.3389/fmed.2023.992493

**Published:** 2023-02-03

**Authors:** Liwei Ying, Guoping Cai, Zhong Zhu, Guoliang Yu, Yongwei Su, Hua Luo

**Affiliations:** ^1^Department of Orthopedics, Taizhou Hospital of Zhejiang Province Affiliated to Wenzhou Medical University, Taizhou, China; ^2^Department of Intensive Care Unit, Taizhou Hospital of Zhejiang Province Affiliated to Wenzhou Medical University, Taizhou, China; ^3^Department of Orthopedics, The First Affiliated Hospital of Jinzhou Medical University, Jinzhou, China

**Keywords:** pronator quadratus, distal radius fractures, fixation, meta-analysis, wrist function

## Abstract

**Introduction:**

The study aimed to evaluate the efficacy of pronator quadratus (PQ) repair versus no repair following volar plate fixation of distal radius fractures.

**Methods:**

A comprehensive search was performed in PubMed, CNKI, EMBASE, Web of Science, Ovid, and Cochrane Library databases. All randomized controlled trials comparing PQ repair with no repair in distal radius fractures before January 2023 were included. Two investigators independently screened eligible articles, assessed the study quality, and extracted data from included studies. Continuous variables used standardized mean difference and 95% confidence interval as efficacy statistics. The meta-analysis was performed using the Revman 5.4 software.

**Results:**

A total of 430 patients in 7 RCT studies were included in this meta-analysis, of which 218 underwent PQ repair, while 212 patients underwent no repair. The results of the meta-analysis displayed statistically significant differences in grip strength (short-term), pronation angle (short-term), and pronation strength (short- and long-term) between the two groups. No significant difference in other outcomes was found between the two treatment arms.

**Discussion:**

The repair of PQ may further increase grip strength and pronation function in the short-term and enhance long-term pronator muscle strength compared to no repair. However, due to the small number of articles included in the study, the above conclusions need to be verified by a larger sample and multi-center clinical study.

## Introduction

Distal radius fracture is one of the most common fractures, accounting for about 17.5% of total body fractures, and the incidence in the elderly population has been increasing these decades (1, 2). Volar locking plate internal fixation has become the primary surgical method for the action of distal radius fractures for its better rehabilitation effect, firmer fixation, and fewer complications (3). However, during the surgery, it is often necessary to cut the pronator quadratus (PQ) muscle near its radial attachment and ulnar retraction to expose the fracture site for better reduction and fixation (4). Due to the poor tissue condition of the PQ muscle and the difficulty of suturing, some clinicians choose not to suture the PQ after fixation of the distal radius fracture (5). At the same time, they also believe that the tight suture of the PQ may lead to postoperative ischemic contracture of the wrist joint, thereby reducing the postoperative range of motion of the wrist joint (6). However, some clinicians believe that suturing the PQ can help patients restore more forearm pronation function after surgery and avoid long-term friction between the volar flexor tendon and the plate (7–9). At the same time, it can also make the inferior radioulnar joint more stable. Therefore, whether to suture the severed pronator quadratus muscle during internal fixation of distal radius fractures remains controversial. Hence, we compare the curative effect of repair and no repair of PQ after volar plate fixation of distal radius fracture by meta-analysis.

## Patients and methods

This meta-analysis was conducted in agreement with the PRISMA statement (10), and the protocol for this study was registered on PROSPERO (Registration No: CRD42022306135).

### Search strategy

We searched all published articles in PubMed, CNKI, EMBASE, Web of Science, Ovid, and Cochrane Library databases using the terms “distal radius,” “volar plating,” and “pronator quadratus” before January 2023. No language restriction was applied. The reference lists of articles retrieved from the electronic were searched for related articles. Two investigators (GLY and YWS) independently performed the search and data extraction process. If there are disagreements about the eligibility of a study, a senior researcher decides after discussion. Questions encountered in the literature or lack of relevant data should be resolved by contacting the original author.

### Inclusion and exclusion criteria

Inclusion criteria: (1) randomized controlled trial (RCT); (2) Population: Distal radius fractures requiring surgery in adult patients; (3) Intervention: The dissected pronator quadratus muscle was repaired with no repair following fixation of distal radius fractures; (4) Outcome: Evaluating the postoperative function of the wrist after surgery.

Exclusion criteria: (1) case reports, reviews, and republished works; (2) Studies not reporting relevant data and contacting the original author to get the data failed; (3) No control group was established.

### Data extraction and study assessment

Two researchers (LY and ZZ) independently extracted all related data from selected studies. Data extracted included the author’s name, year of publication, country, sample size, ages in years, plate type, suture type, study outcomes, and follow-up time. The Cochrane Collaboration risk assessment tool was used to assess the risk of bias. In case of disagreement, the disagreement is resolved through discussion or negotiation by a third researcher.

### Statistical analysis

The Meta-analysis was performed using RevMan 5.4 software (Cochrane Collaboration, Oxford, UK). Continuous variables used standardized mean difference (SMD) and 95% confidence interval (CI) as efficacy statistics. When there is no statistical homogeneity among the pooled studies (*p* > 0.1, I^2^ < 50%), a fixed-effect model is used; when there is statistical heterogeneity among the pooled studies (*p* ≤ 0.1 or I^2^ ≥ 50%), the random-effect model should be used. The source of heterogeneity was analyzed, and if the reason for the heterogeneity could not be found, the random-effects model was used for analysis. DASH (Disabilities of the Arm, Shoulder and Hand) and QuickDASH scores were combined for the meta-analysis. For studies not reporting mean and standard deviation scores of outcome variables, the same was estimated based on median and range using methods informed by Wan et al. (11). Finally, draw a publication bias funnel plot to qualitatively evaluate whether there is publication bias in the statistical data. *p* < 0.05 was considered a significant difference.

## Result

### Study characteristics

**Figure 1:**
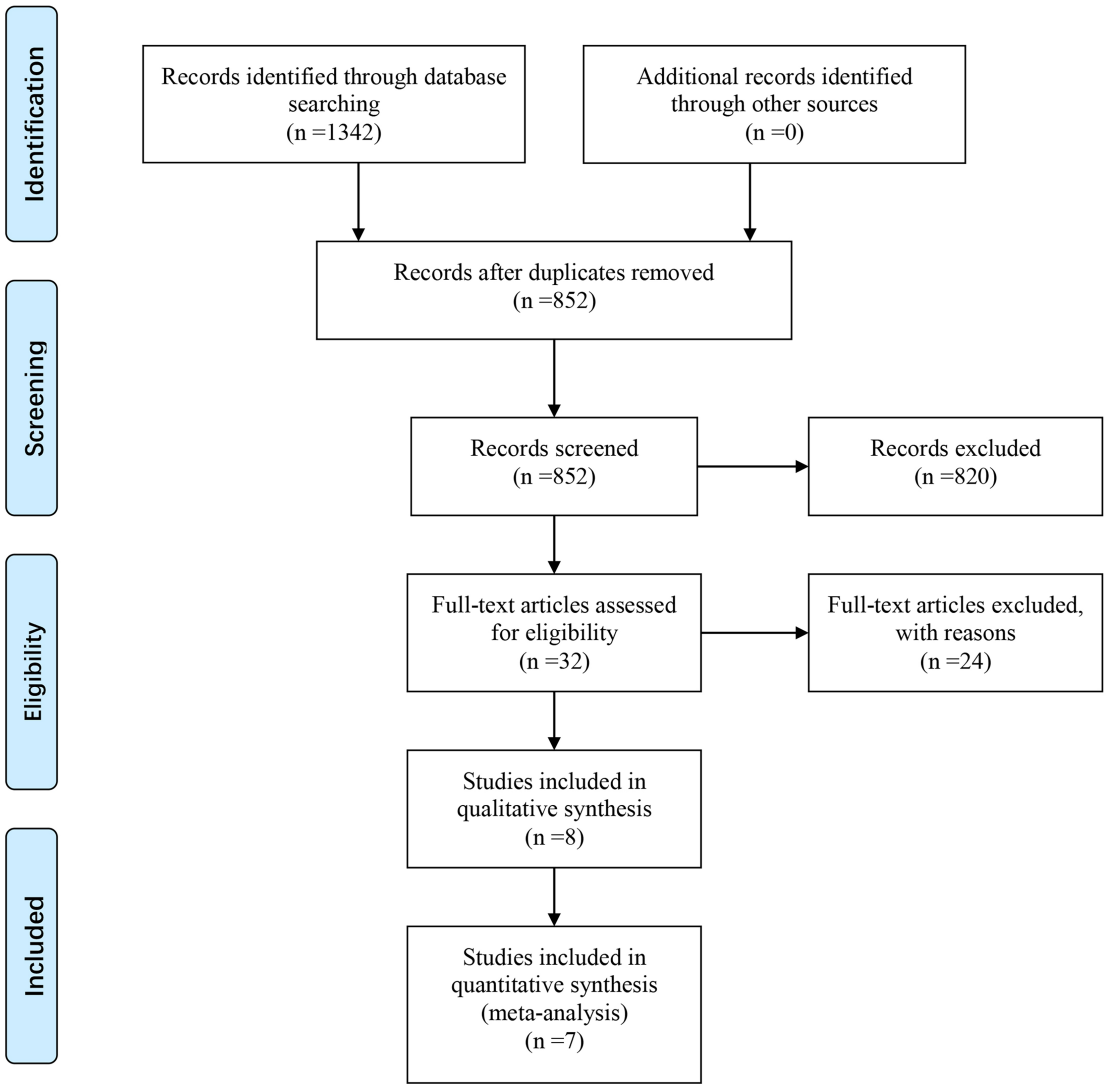
Flow diagram for search and selection of included studies.

**Figure 2:**
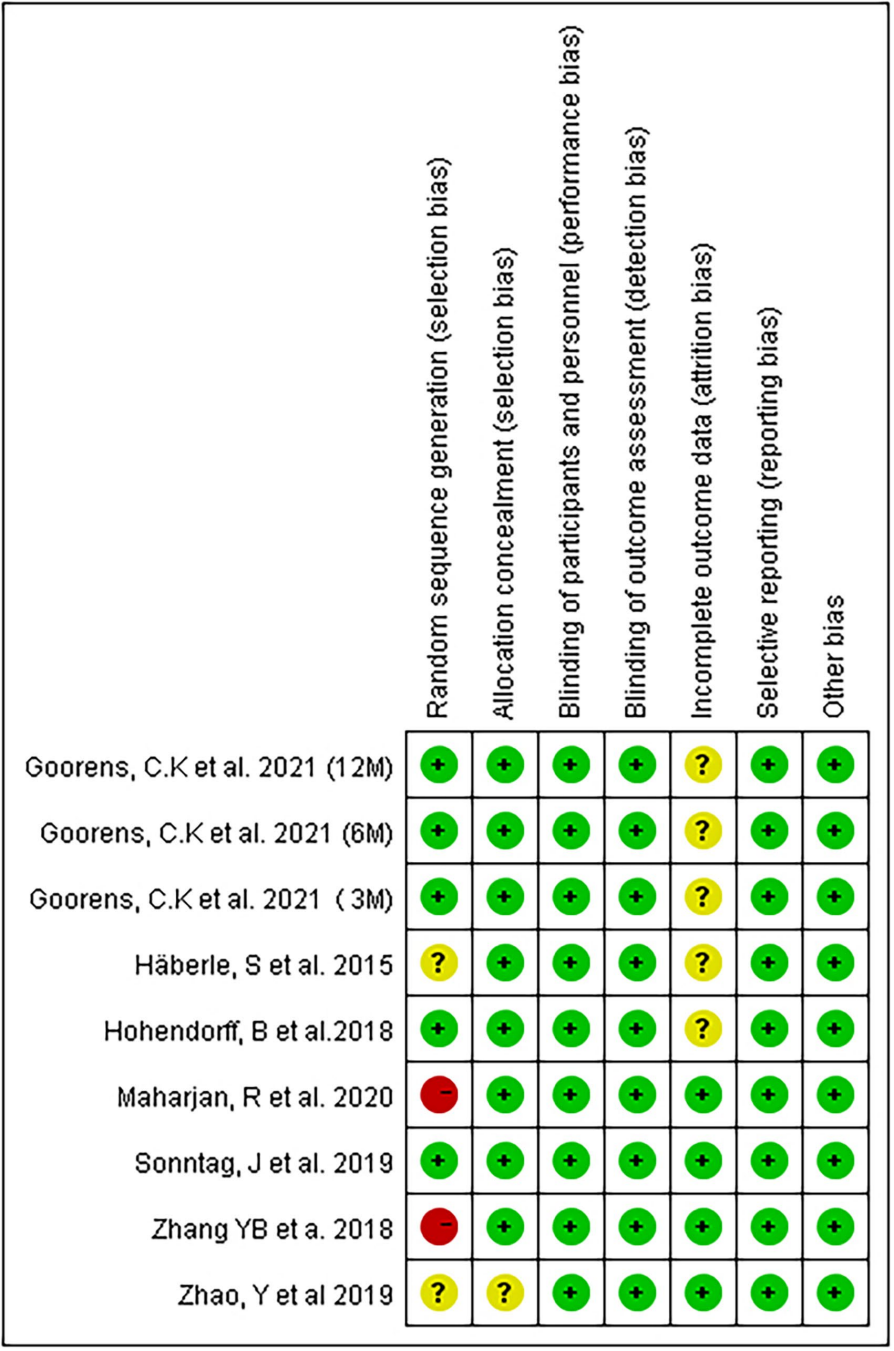
Risk of bias summary: review authors’ judgments about each risk of bias item for each included study.

A total of 1,342 articles were retrieved by searching the above databases. Two researchers independently conducted the literature screening process according to the inclusion and exclusion criteria. Four hundred ninety duplicate articles were eliminated, and 820 irrelevant documents were eliminated by reading the titles and again. Of the remaining 32 studies read in full text, 24 were excluded. Among these 24 studies, 3 RCT registries lacked results, 2 registered RCTs were published online, 1 meeting report, 2 lacked outcomes, 8 retrospective studies, and 8 studies missing wrist function. Finally, eight articles were included in the systematic review (6, 12–18). One of the studies lacked relevant statistical data (12), and we failed to request the data by email. Unfortunately, the study had to be excluded. Finally, 7 RCT studies registering 430 patients were included in this meta-analysis (6, 13–18). The literature screening flowchart is shown in Figure 1. The risk of bias assessment results is shown in Figure 2. Table 1 provides the information of the include studies.

**Table 1:** Characteristics of included studies.

Study (author, year)	Country	Study type	Sample size		Ages in years		Plate type used	Suture type used	Follow-up (month)
			Repair	No repair	Repair	No repair			
Goorens, 2021	Belgium	RCT	35	30	59.5 ± 21.9	63.8 ± 20.8	Synthes VLP	Vicryl 3–0	12
Häberle, 2015	Germany	RCT	31	29	NS	NS	Medartis (VA) and Synthes (VA) VLP	Vicryl 3–0	3
Hohendorff, 2018	Germany	RCT	20	16	NS	NS	Stryker (VA) VLP	PDS 4–0	12
Maharjan, 2020	Nepal	RCT	13	19	32.0 ± 15.58	27.68 ± 8.38	Unknown VLP	Absorbable 2–0	12
Sonntag, 2019	Denmark	RCT	32	31	62.0 ± 10.8	63.6 ± 15.6	Stryker (VA) and Synthes (VA)	Vicryl 3–0	12
Zhang, 2018	China	RCT	45	45	55.7 ± 3.4	54.1 ± 2.4	Unknown VLP	Absorbable 2–0	6
Zhao, 2019	China	RCT	42	42	48.7 ± 7.7	47.1 ± 8.5	NS	Absorbable 3–0	3

RCT, randomised controlled trial; NS, Not specified; VLP, volar locking plate; VA, variable angle; PDS, polydioxanone.

### Meta-analysis of the outcomes

**Figure 3:**
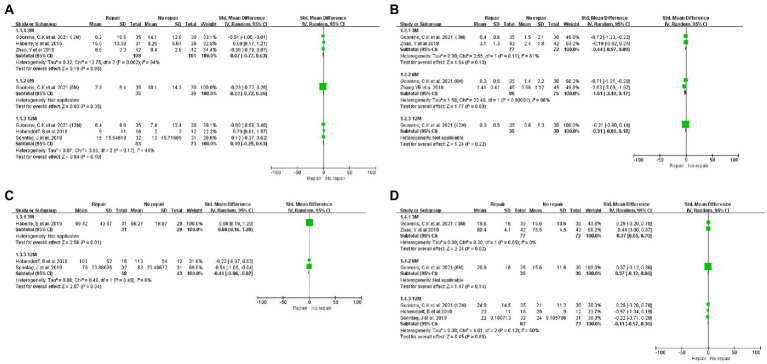
Forest plots of the DASH **(A)**, pain score **(B)**, pronation strength **(C)**, and grip strength **(D)** between the two treatment arms.

**Figure 4:**
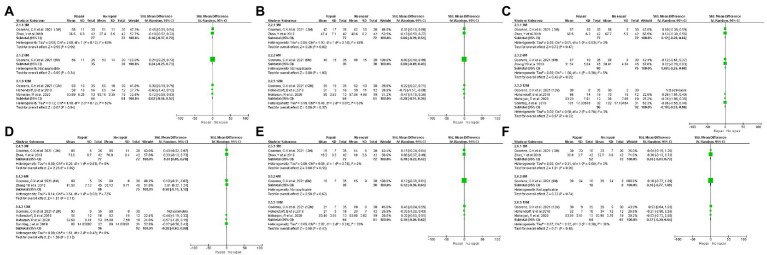
Forest plots of the SMD in wrist mobility. Forest plots of the SMD in wrist mobility between the two treatment arms: extension **(A)**, flexion **(B)**, supination **(C)**, pronation **(D)**, radial deviation **(E)**, and ulnar deviation **(F)**.

One study analyzed short-term (3 M), mid-term (6 M), and long-term (12 M) outcomes in patients with or without PQ repair, and we divided this study into three sub-studies for statistical analysis (16). We retrieved each study’s short-term, mid-term, and long-term follow-up outcomes. Figures 3, 4 showed forest plots of different clinical outcomes between the two groups. There were statistically significant differences between the repaired group and the no repaired group for pronation strength at short- (SMD: 0.68, 95% CI: 0.16 to 1.20, *p* = 0.01) and long-term (SMD: -0.44, 95% CI: −0.86 to −0.02, *p* = 0.04)of follow-up (Figure 3C). Statistical significance was found for grip strength during the short-term follow-up (SMD: 0.37, 95% CI: 0.05 to 0.70, *p* = 0.02; Figure 3D). The changes in the pronation angle of the patients were statistically significant during the short-term follow-up (SMD: 0.37, 95% CI: 0.05 to 0.70, *p* = 0.02), but there was no difference during the mid-term (SMD: 0.50, 95% CI: −0.11 to 1.12, *p* = 0.11)and long-term follow-up (SMD: −0.28, 95% CI: −0.63 to 0.08, *p* = 0.13; Figure 4D). No significant difference in other outcomes was found between the two treatment arms.

### Publication bias

Considering the small sample size (< 10) in our meta-analysis, funnel plot analysis was not applicable for determining publication bias.

## Discussion

Our results showed that the changes in grip strength and pronation function of the pronator quadratus muscle group after volar plate fixation for distal radius fractures were better than those of the control group during the short-term follow-up. Still, there was no statistical significance in the mid-term and long-term follow-ups. Statistical significance was found for pronation strength at short- and long-term follow-ups. There were no significant differences in DASH score, pain score, and wrist range of motion (except pronation) in the short-term, mid-term and long-term follow-ups. Theoretically, the pronator is the primary muscle responsible for pronation in the forearm, and if it is not repaired, it can lead to loss of wrist function. Our study showed that the pronation function and grip strength of the pronator anterior muscle after repair were better than those of the control group in the short term. The pronator muscle strength was superior to the control group. Based on our findings, we speculate that the suture of the PQ muscle has little effect on the wrist joint’s mid and long-term function after surgery for distal radius fractures. In other words, to a certain extent, the pronator quadratus muscle itself may play a relatively minor role in wrist joint function, so there is no significant difference in the wrist joint function after the pronator muscle is severed during the operation in the long-term follow-up. Due to the limited number of articles in the article, the above conclusions need to be verified by a larger sample and multi-center clinical study.

The pronator quadratus muscle is located deep in the forearm, and its primary function is pronation, occasionally used for tissue reconstruction (19). Scholars have found that the PQ contributes about 21% of the pronation torque during pronation and grasping (20). Due to the lack of fascia coverage on the surface, it is easy to tear after repairing PQ. The indwelling of the plate further increases the suture tension and the difficulty of the suture (21). The muscle of the brachioradialis and pronator quadratus are embedded in each other. Complete separation of the two muscles during surgery may allow for better suturing of the PQ (22). Wu et al. found that intraoperative reconstruction of the PQ can reduce early postoperative pain in patients (23). However, the repaired PQ does not function well because of the poor quality of surrounding muscle and fascial tissue, which may cause pain and hinder pronation and supination of the forearm after repair (24). McConkey et al. informed that after pronator resection, pronation may result in a pronation torque deficit (25). And repairing the PQ to cover the internal fixation plate can further avoid complications such as tendon rupture or flexor tendon wear. Therefore, repairing the PQ can theoretically further preserve the mobility of the wrist joint, protect the flexor tendons from the sharp edges of the volar plate and screws, and act as a “dynamic stabilization device” for the distal radioulnar joint (8, 26, 27). However, some scholars have shown that it is still controversial whether repairing the PQ is beneficial to stabilizing the distal radioulnar joint (28). Fang et al. found that the patients removed the plate after fracture healing, 23 patients had apparent atrophy of the PQ after repair, and prominent muscle scar and fibrosis. There was no difference in hand function between these 23 patients and other patients (29). Spies et al. (30) have shown that patients with late tendon irritation after PQ repair persist, mainly where flexor pollicis longus rupture was caused by the plate being positioned too far, which may increase the risk of tendon injury.

A meta-analysis by Shi et al. (31) reported that the PQ might not be necessary to be repaired after fixation of distal radius fractures. One of the RCT studies included in this meta-analysis has not mentioned relevant statistical data (12), the author only estimated its mean and standard deviation through the chart in the text. Besides, the author has not performed a subgroup analysis and mistakenly combined the results of 3 months and 12 months for meta-analysis. Besides, there was no statistical difference in wrist function between the repair group and the non-repair group was found in their study. Lu et al. performed a meta-analysis and found that PQ muscle repair showed different effects on pronation strength in different groups (32).We conducted a subgroup analysis by the length of follow-up time and the results showed that the significant difference in pronation angle between the two groupsafter short-term and long-term follow-up. Both previous articles included some retrospective studies, and the level of evidence was low. In our meta-analysis, all included studies were RCT, subgroup analysis was performed according to the length of follow-up, and articles with no data were excluded. The level of evidence is higher and more scientific.

## Limitation

This review carries potential limitations. First, the sample size of this study is small, only 7 RCT studies are included in our study. Secondly, the follow-up time of each study is different, resulting in a smaller sample size for each group included in the subgroup analysis. Thirdly, due to the lack of original data in these included studies, we have not performed a subgroup analysis according to the fracture classification. Different fracture classifications have different postoperative wrist functions. Fourthly, considering that the PQ muscle is prone to tear after repair, the compensatory function of the deep head of the PQ and the pronator teres muscle may affect the results. In addition, studies have shown that both repaired and unrepaired PQ may have scarred during follow-up, and the difference between the two is not sufficient to affect the outcome measures (33).

## Conclusion

Combined repair of pronator quadratus muscle based on internal fixation for distal radius fractures may further increase grip strength and pronation function in the short-term, as well as enhance long-term pronator muscle strength. However, due to the small number of articles included in the article, the above conclusions need to be verified by a larger sample and multi-center clinical study.

## Data availability statement

The original contributions presented in the study are included in the article/Supplementary material, further inquiries can be directed to the corresponding author.

## Author contributions

LY collected and analyzed the data. ZZ made substantial contributions to the analysis, prepared, and revised the manuscript. GC revised and approved the manuscript. GY and YS performed the literature retrieval and drafted the article. HL designed the meta-analysis, collected and analyzed the data, and wrote the first draft of the manuscript. All authors contributed to the article and approved the submitted version.

## Conflict of interest

The authors declare that the research was conducted in the absence of any commercial or financial relationships that could be construed as a potential conflict of interest.

## Publisher’s note

All claims expressed in this article are solely those of the authors and do not necessarily represent those of their affiliated organizations, or those of the publisher, the editors and the reviewers. Any product that may be evaluated in this article, or claim that may be made by its manufacturer, is not guaranteed or endorsed by the publisher.
